# Photo-induced enhanced Raman spectroscopy as a probe for photocatalytic surfaces

**DOI:** 10.1098/rsta.2022.0343

**Published:** 2023-10-30

**Authors:** Sultan Ben-Jaber, Daniel Glass, Thomas Brick, Stefan A. Maier, Ivan P. Parkin, Emiliano Cortés, William J. Peveler, Raúl Quesada-Cabrera

**Affiliations:** ^1^ Department of Chemistry, University College London, 20 Gordon Street, London WC1H 0AJ, UK; ^2^ Department of Science and Forensics, King Fahad Security College, Riyadh, Saudi Arabia; ^3^ The Blackett Laboratory, Department of Physics, Imperial College London, London SW7 2AZ, UK; ^4^ School of Physics and Astronomy, Monash University, Clayton, Victoria 3800, Australia; ^5^ Chair in Hybrid Nanosystems, Faculty of Physics, Ludwig Maximilians Universität München, 80799 München, Germany; ^6^ School of Chemistry, Joseph Black Building, University of Glasgow, Glasgow G12 8QQ, UK; ^7^ Department of Chemistry, Institute of Environmental Studies and Natural Resources (i-UNAT), Universidad de Las Palmas de Gran Canaria, Campus de Tafira, Las Palmas de GC 35017, Spain

**Keywords:** photo-induced enhanced Raman spectroscopy, oxygen vacancies, surface-enhanced Raman spectroscopy, defected titanium dioxide, photocatalysis

## Abstract

Photo-induced enhanced Raman spectroscopy (PIERS) has emerged as a highly sensitive surface-enhanced Raman spectroscopy (SERS) technique for the detection of ultra-low concentrations of organic molecules. The PIERS mechanism has been largely attributed to UV-induced formation of surface oxygen vacancies (V_o_) in semiconductor materials, although alternative interpretations have been suggested. Very recently, PIERS has been proposed as a surface probe for photocatalytic materials, following V_o_ formation and healing kinetics. This work establishes comparison between PIERS and V_o_-induced SERS approaches in defected *noble-metal-free* titanium dioxide (TiO_2-*x*_) films to further confirm the role of V_o_ in PIERS. Upon application of three post-treatment methods (namely UV-induction, vacuum annealing and argon etching), correlation of V_o_ kinetics and distribution could be established. A proposed mechanism and further discussion on PIERS as a probe to explore photocatalytic materials are also presented.

This article is part of the theme issue ‘Exploring the length scales, timescales and chemistry of challenging materials (Part 2)’.

## 1. Introduction

Surface-enhanced Raman spectroscopy (SERS) is a powerful analytical technique with broad-ranging applications, including chemical and biochemical sensing, electrochemistry and catalysis [1]. In conventional SERS, the Raman signal of molecules adsorbed onto appropriate surfaces may be enhanced by several orders of magnitude [2–7]. The mechanism of SERS has been explained on the basis of two combined processes, an electromagnetic (EM) effect and a chemical enhancement (CE) contribution [8–10]. The former is usually the main contribution to SERS and it is induced by resonant excitation of electron oscillations (plasmons) within metal nanoparticles [11]. In the CE mechanism, the Raman cross-section of a molecule is enhanced due to changes in its electronic polarizability upon adsorption to a substrate or upon resonant charge-transfer events between the adsorbed molecule and the substrate [12].

Some years ago, our group reported on band enhancement beyond that of conventional SERS using an effect we termed as photo-induced enhanced Raman spectroscopy, or PIERS [13]. In our PIERS experiments, hybrid noble metal-supported titanium dioxide (TiO_2_) substrates were irradiated under prolonged UV light prior to the deposition of an organic adsorbate, used as target molecule. Band enhancement was attributed to an interaction between the noble metal-molecule-semiconductor system and photogenerated oxygen vacancies (V_o_) at the semiconductor surface [14]. Photogenerated V_o_ states introduce donor sites at approximately 0.8 eV below the conduction band (CB) edge of TiO_2_, [15] which may allow for the promotion of electrons into the CB under laser irradiation. These electrons can then transfer into the Fermi level of noble-metal nanoparticles and return to V_o_ states, strengthening the CE contribution in the Raman signal. Formation of surface V_o_ sites under UVC-light irradiation (*λ *= 254 nm) was later confirmed via time-resolved atomic force microscopy [16]. The proposed mechanism based on V_o_ was also supported by the fact that PIERS is a transient effect and the enhanced signals decreased within a range of 30–60 min upon exposure to air, which was attributed to the loss of surface vacancies [17].

The use of PIERS as a sensing technique has been reviewed recently [18]. As it has been pointed out, different operation procedures have been followed, either *ex situ*—with irradiation of the substrate before or after deposition of the target molecule—or *in situ*, with irradiation during the Raman analysis. PIERS studies have been applied using a wide range of substrate materials, from single semiconductors to hybrid noble metal-insulators. The influence of key parameters, such as irradiation conditions (wavelength, irradiance, illumination time) or substrate structure and morphology, has been discussed using a range of target adsorbates. Most of these studies have explained the PIERS mechanism based on the formation of oxygen vacancies, as indicated in the recent review and references therein [18]. Other authors have questioned the formation of oxygen vacancies and attributed the PIERS effect to photo-induced charge-transfer (CT) processes [19–22]. Interestingly, in contrast to previous observations, a very recent work demonstrated a long-term PIERS effect (greater than 8 days) using gold-embedded porous TiO_2_ films as PIERS substrates [23]. Based on cathodoluminescence measurements, these authors proposed two different mechanisms for PIERS depending on the arrangement of the noble-metal nanoparticles, either supported on or embedded in the substrate. The former would be supported by the presence of oxygen vacancies, as conventionally accepted, while the latter is due to a back transfer of charges related to plasmon-induced charge separation in Schottky barriers with a narrow depletion zone [23].

Recently, the PIERS effect has been used as a surface probe to evaluate photocatalytic materials [24]. UV-induced V_o_ dynamics were monitored *in situ*, following decay kinetics of Raman enhancements upon healing of V_o_ sites. Using different noble metal-supported metal-oxide substrates, correlation was established between V_o_ kinetics and photocatalytic properties, showing the key role of induced V_o_ lifetimes in photocatalytic performance. This novel application of PIERS comes as a *user-friendly* complementary tool to specialized techniques such as scanning tunnelling microscopy (STM) and electron paramagnetic resonance (EPR). A very recent study, based on EPR and *in situ* infrared spectroscopy, has confirmed the role of V_o_ as dynamic active sites in photocatalytic reactions, via activation of oxygen molecules [25]. A question then remains whether PIERS can be applied as a tool for the evaluation of generic photocatalytic substrates and under conditions relevant to photocatalytic applications.

In this work, the PIERS effect is used for the monitoring of V_o_ kinetics in defected *noble metal-free* TiO_2-*x*_. In the case of noble metal-free SERS substrates, Raman enhancements can mostly be attributed to CE mechanisms. Interesting emerging materials in this group include two-dimensional inorganic compounds (MXenes) [22] and transition metal dichalcogenides [26], in addition to simple metal-oxide (M_X_O_Y_) semiconductors, such as Cu_2_O, [27] W_18_O_49_ [28] and TiO_2_, [29,30], amongst others. The exact proportion of the two elements (M and O) in the latter group provides a degree of freedom for the enhancement of the CE factor in SERS. The engineering of defected metal oxides with oxygen vacancies (V_o_) has been used to stimulate charge-transfer processes across a wide range of applications [31–33]. Here, the generation of V_o_ in TiO_2_ substrates is induced upon reduction of Ti^4+^ to Ti^3+^ species via UV-light irradiation, vacuum annealing or inert-gas etching treatment [32,34]. Dynamic Raman studies monitoring V_o_ kinetics are followed indirectly here, using a model molecule for SERS (rhodamine-6G) over post-treated TiO_2-*x*_ substrates. Comparison is established among the three treatment methods and the implications to the PIERS mechanism are discussed. Further discussion is also presented towards the use of PIERS in photocatalysis.

## 2. Methods

### (a) Synthesis of TiO_2_ thin films

All chemicals were purchased from *Sigma-Aldrich* and used as received. TiO_2_ thin films were synthesized using atmospheric-pressure chemical vapour deposition (CVD) from titanium tetrachloride (TiCl_4_, 99%) and ethyl acetate (EtAc or C_4_H_8_O_2_, 99.8%) as metal and oxygen precursors, respectively. All components of the CVD apparatus were kept at high temperature (200°C). The precursors were mixed in a stainless-steel chamber (250°C) before accessing the CVD reactor, which consisted of a 320 mm-long graphite heating block fitted in a quartz tube with three Whatman heater cartridges. The temperature of the system was controlled by Pt-Rh thermocouples. Nitrogen (*BOC*) was used as the carrier gas. Precursor bubbler temperatures and gas flows were set to 1.2 l min^−1^ 70°C and 0.25 l min^−1^ 40°C for TiCl_4_ and EtAc, respectively. The TiO_2_ films were deposited at 500°C on quartz slides (25 × 25 mm, *Multi-Lab*). Typical growth rates under these conditions were *ca* 0.3 µm min^−1^. Film thicknesses (*ca* 500 nm) were estimated using the Filmetrics F20 machine operating in reflectance mode in air against a FTO standard.

### (b) Substrate preparation

Rutile TiO_2_ films were obtained from thermal treatment of as-deposited anatase films to 1000°C for 5 h in air. Preliminary SERS studies were carried out on the annealed substrates and used as reference. The rutile substrates were then treated following either (a) UV-light irradiation for 2 h; (b) vacuum annealing at 1000°C for 1 h; (c) argon etching under high vacuum using argon ion bombardment in the XPS instrument (details below). A germicidal lamp (UVItec LI 215G) was used for UV treatment (UVC light, *λ* = 254 nm, 12 mW cm^−2^). Reference data were obtained from films annealed under air or etched and subsequently healed during exposure to air for 3 days. The Raman measurements were carried out using rhodamine-6G (R6G) dye as target molecule, which was drop-casted onto the TiO_2_ substrates from a 10^−5^ M methanol solution.

### (c) Characterization techniques

Raman studies were carried out using a Renishaw 1000 spectrometer equipped with a He-Ne laser (*λ *= 633 nm) and coupled to a microscope with a 50× objective. The Raman system was calibrated using a silicon reference. Acquisition time was 10 s. Raman scattering spectra were recorded in the range of 150–2000 cm^−1^. X-ray diffraction (XRD) analysis was carried out using a Bruker AXS D8 (Lynxeye XE) diffractometer with a monochromated copper X-ray source (K*α*_1_, *λ* = 1.54 Å) under a glancing incident angle (*θ*) of 1°. UV–Vis spectroscopy was performed using a Perkin Elmer Lambda 950 UV/Vis/NIR Spectrophotometer. A Labsphere reflectance standard was used as a reference. X-Ray photoelectron spectroscopy (XPS) was performed using a Thermo K-α spectrometer with monochromated aluminium K*α* radiation, a dual beam charge compensation system and constant pass energy of 50 eV. Survey scans were collected in the range of 0–1200 eV. High-resolution peaks were used for the principal peaks of Ti *2p*, O *2p* and C *1s*. The peaks were modelled using sensitivity factors to calculate the film composition. The area underneath these bands is an indication of the element concentration within the region of analysis (spot size: 400 µm).

## 3. Results and discussion

**Figure 1.**
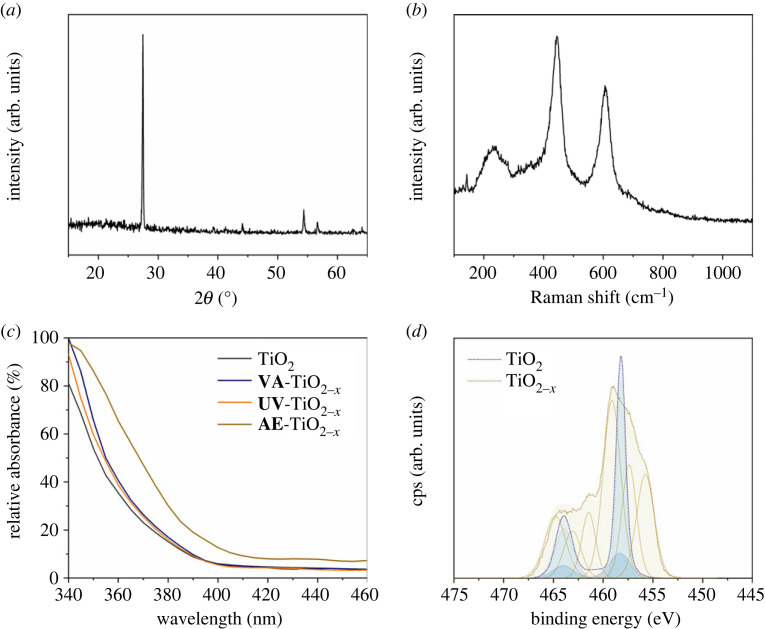
Characterization of TiO_2_ substrates. (*a*) X-ray diffraction and (*b*) Raman spectra of a typical rutile TiO_2_ film. (*c*) Absorption spectra of TiO_2-*x*_ films after post-treatment using UVC-light irradiation (**UV-**TiO_2-*x*_), vacuum annealing (**VA-**TiO_2-*x*_) and argon etching (**AE-**TiO_2-*x*_). The spectrum of an as-prepared TiO_2_ film is included for reference. (*d*) XPS Ti *2p* region before (blue lines) and after (orange lines) argon etching (**AE-**TiO_2-*x*_).

Post-treated rutile TiO_2-*x*_ films (500 nm) were used as PIERS substrates, following dynamic Raman studies of R6G dye. X-ray diffraction and Raman spectroscopy analysis confirmed the presence of the rutile phase (figure 1*a,b*). The post-treatment methods included (a) prolonged irradiation under UVC light; (b) vacuum annealing; and (c) argon etching under high vacuum conditions (see Methods)—henceforth, **UV-**TiO_2-*x*_, **VA-**TiO_2-*x*_ and **AE-**TiO_2-*x*_ films, respectively. In every case, the post-treatment of the films resulted in a blue coloration of the films, which was attributed to the reduction of Ti^4+^ into (blue) Ti^3+^ species. The coloration was particularly intense in the case of **AE-**TiO_2-*x*_ films, whereas it was comparably faint in the other two cases. These observations were consistent with corresponding changes in UV/Vis absorbance (figure 1*c*). A red shift of the absorbance was significant in the case of etched films while it was less pronounced for **UV-**TiO_2-*x*_ and **VA-**TiO_2-*x*_ films. Similar changes in optical properties have been attributed to the formation of sub-levels in the bandgap of TiO_2_ due to formation of oxygen vacancies (V_o_) [35]. It is worth noticing that UV/Vis spectroscopy is a bulk technique and the formation of small densities of surface V_o_ sites may not have a drastic impact on absorbance. Previous studies [36] have noticed the emergence of a broad band centred around *ca* 525 nm upon formation of V_o_ sites. Unfortunately, interference in our optical measurements (so-called *Newton* rings) hindered the unequivocal detection of this band. Both vacuum annealing and UVC-light irradiation are known to produce small densities of Ti^3+^ species at the catalyst surface [37]. On the other hand, argon etching is known to form significant defect states in metal-oxide materials. The presence of reduced Ti^3+^ species could be confirmed for **AE-**TiO_2-*x*_ films in the Ti *2p* environment using XPS (figure 1*d*). These changes were reversible upon exposure to air, consistent with previous observations [38].

**Figure 2.**
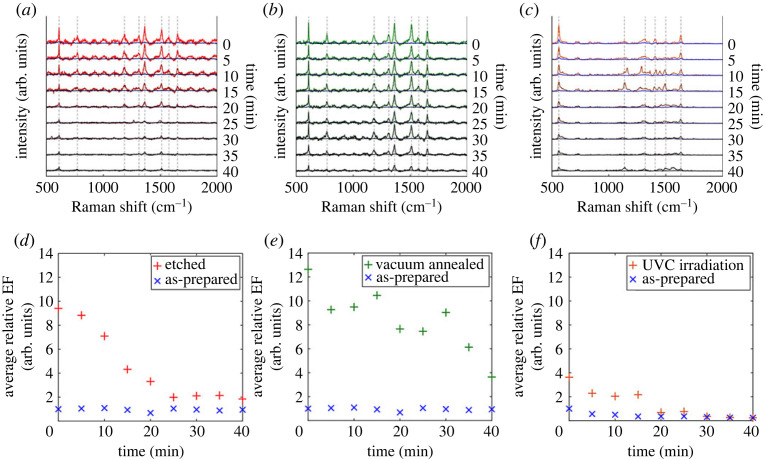
Dynamic Raman studies of R6G dye deposited on post-treated TiO_2_ films upon exposure to air. The post-treatment processes included (*a*) argon etching, **AE-**TiO_2-*x*_ (red lines); (*b*) vacuum annealing, **VA-**TiO_2-*x*_ (green lines); and (*c*) UVC-light irradiation, **UV-**TiO_2-*x*_ (orange lines). Corresponding average enhancement factors (EFs) were obtained relative to SERS spectra (blue symbols) using the untreated films (*d–f*).

Dynamic Raman studies of R6G were carried out upon deposition of the dye immediately after the post-treatment of the TiO_2_ substrates. R6G was chosen as model molecule due to its high stability and Raman cross-section [39]. The three sets of data are shown in figure 2*a–c*. In the first instant, right after deposition of the dye (*t* = 0 min), considerable enhancement of the Raman bands was observed for the three post-treated TiO_2-*x*_ films with respect to similar SERS analysis before treatment (figure 2). The Raman spectrum of R6G is characterized by eight main vibrational modes, with an additional band at 611 cm^−1^ due to overlapping of in-plane C–C–C ring bending vibrations and the TiO_2_ A_1g_ mode [39]. The enhancement of the R6G spectra adsorbed to the TiO_2-*x*_ substrates was band-selective, which is a typical fingerprint of the CE in SERS [13]. Average enhancement factors (EFs), collected from 24 to 30 positions across the sample, relative to those obtained on the untreated films, were estimated as ×4.61, ×5.58 and ×7.61, respectively, for **UV-**TiO_2-*x*_, **AE-**TiO_2-*x*_ and **VA-**TiO_2-*x*_ films (table 1). This result may seem surprising considering that the **AE-**TiO_2-*x*_ film was defect-rich compared with **UV-**TiO_2-*x*_ and **VA-**TiO_2-*x*_ films. Nonetheless, it is consistent with previous observations establishing an optimum V_o_ density with a peak in photocatalytic efficiency [24]. V_o_ densities must be high enough to favour carrier transport but not as high as to promote carrier trapping. A similar analogy may be followed in road transport, where enough vehicles help people move around easily but too many cause people to get stuck in traffic jams.

**Table 1.** Average enhancement factors (EFs) of R6G Raman bands using post-treated TiO_2_ substrates, namely UVC-light irradiated (**UV-**TiO_2-*x*_), argon-etched (**AE-**TiO_2-*x*_) and vacuum-annealed (**VA-**TiO_2-*x*_) films. EF values were obtained relative to SERS enhancements on the same films before treatment. Errors account for the average range upon surface mapping of the films. Calculated V_o_ healing lifetimes were obtained upon exposure of post-treated films to air.

sample treatment	relative EF	V_o_ healing lifetime (min^−1^)
TiO_2_	1 ± 0.45	
UV-TiO_2-*x*_	4.61 ± 0.36	16.69 ± 0.73
AE-TiO_2-*x*_	5.58 ± 2.07	18.00 ± 0.49
VA-TiO_2-*x*_	7.61 ± 2.10	209.16 ± 72.54

A series of Raman spectra were recorded after deposition of the dye and every 5 min at a single position on the TiO_2-*x*_ films. A clear intensity decrease was continuous for about 40 min from the initial enhanced spectra (PIERS), eventually reaching average SERS intensities. These trends over and above any laser-induced photobleaching are highlighted in figure 2*d–f* [17]. Band decrease rates followed the same trend of average EFs, i.e. intensities dropped fast on **UV-**TiO_2-*x*_ films, followed by **AE-**TiO_2-*x*_ and **VA-**TiO_2-*x*_ films. Induced V_o_ healing lifetimes for each treatment were estimated following a method reported in previous work (table 1). The longest V_o_ lifetimes corresponded to **VA-**TiO_2-*x*_ films, which remained blue for a long period of time compared with **UV-**TiO_2-*x*_ and **AE-**TiO_2-*x*_ films. This observation was in spite of the initial dramatic coloration of the **AE-**TiO_2-*x*_ and it was attributed to the potential presence of bulk V_o_ sites in **VA-**TiO_2-*x*_ films. Oxygen atoms may diffuse from the bulk of the sample, particularly under vacuum annealing conditions, causing bulk defects [36]. This is particularly relevant to defect engineering strategies in photocatalysis [40], where V_o_ kinetics monitored using PIERS could be used to establish V_o_ distributions under operation conditions.

**Figure 3.**
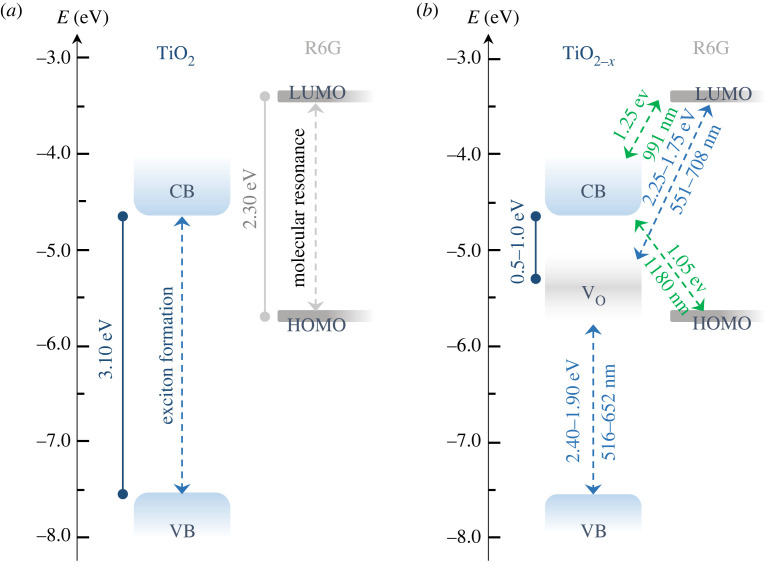
Proposed charge-transfer (CT) pathways in defected TiO_2-*x*_. (*a*) Energy level diagram showing the band structure of TiO_2_ and the HOMO/LUMO of R6G. (*b*) Oxygen-vacancy defects introduce band states at 0.5–1.0 eV below the CB of TiO_2-*x*_. Viable resonant CT pathways open between transient vacancy levels and the VB and the LUMO.

The spectral enhancement observed in TiO_2-*x*_ substrates can be attributed predominantly to semiconductor-analyte charge transfer (CT). The CT mechanism can take place either from V_o_ sites into the CB or from the valence band (VB). The optimum enhancement for CT-type transitions should take place at one of the band edges since CT resonance is at maximum where the density of states varies sharply. The semiconductor can also introduce inter-band transitions, which may contribute to SERS enhancement. These resonances contribute to conventional metal-based SERS in the same mechanistic way as molecular resonances. Thus, inter-band resonances should be considered in addition to CT contributions. Both types, molecular and inter-band resonances, may be close in energy and interfere constructively or destructively. Without V_o_ defect states, the promotion of electrons from the highest occupied molecular orbital (HOMO) to the lowest unoccupied molecular orbital (LUMO) in R6G would require of two consecutive processes: charge transfer from the HOMO into the CB followed by transfer into the LUMO (figure 3). These CT processes involve excitation energies of 1.05 and 1.25 eV, respectively, and they were ruled out under the excitation conditions of our experiments (i.e. photon energy of 1.9 eV, *λ* = 633 nm). Following a semiconductor-to-analyte CT mechanism with participation of V_o_ defect states at 0.5–1.0 eV below the CB, CT resonance may promote electrons from VB into V_o_ and from V_o_ and LUMO (figure 3). Direct excitation within the bandgap of rutile TiO_2_ (*E =* 3.0 eV) and within the HOMO–LUMO gap in the R6G molecule (*E =* 2.30 eV) was ruled out under the laser excitation energy used in our experiments (*E =* 1.9 eV). This was confirmed upon Raman studies using an argon laser source (*E *= 2.4 eV), which promoted the spectrum of R6G (electronic supplementary material, figure S1). The band enhancement observed under low-energy excitation was thus attributed to molecular resonance.

Further work is required, with attention to theoretical studies, to ascertain the underlaying mechanisms behind PIERS. As inferred in previous studies [23], the mechanisms will depend on the type and engineering of substrate materials and target molecules used. As highlighted in the literature [18], the participation of V_o_ in ultrawide-bandgap semiconductors and materials that are not prone to producing vacancies is questionable. Any collateral change upon UV irradiation could affect phonon vibrations, potentially affecting Raman band intensities. Even within materials widely employed in SERS and relevant to photocatalysis such as TiO_2_, the mechanisms of V_o_ formation may follow different pathways depending on the polymorph used. It is known, for instance, that anatase (101) surfaces do not contain surface V_o_ sites, unlike rutile (110) surfaces. [41] PIERS studies using different single crystals can therefore shed light on potential mechanisms for band enhancement.

Close to widespread solar photocatalysis applications, it is also important to investigate PIERS under UVA-light irradiation (*λ *= 365 nm) in the range of a few mW cm^−2^. Following our initial studies [13], PIERS enhancement was confirmed after UVA treatment of rutile TiO_2-*x*_ films using model organic compounds (electronic supplementary material, figure S2). Similar irradiation conditions have been used in the literature [20,42,43]. Aktas *et al*. [43] investigated hybrid Ag-supported TiO_2_ films under UVA light (4.5 mW cm^−2^) during short irradiation periods, noticing correlation between irradiation time and band enhancement until reaching *saturation* (greater than 10 min). Prolonged irradiation beyond that point did not result in any further enhancement.

The role of surface-bound species in the PIERS mechanisms should also be considered. There is extensive literature on the interactions of water and oxygen molecules and V_o_ sites. Dissociation of water molecules, for instance, has been observed in V_o_ sites of rutile TiO_2_ [44]. As mentioned above, one of the ordinary procedures in PIERS experiments is the pre-irradiation of the substrates followed by deposition of the target molecule, typically from solvents such as methanol or ethanol solutions. Organic solvent molecules can act as sacrificial electron donors on TiO_2_ scavenging photogenerated holes and contributing to electron transfer rates [45]. In the case of methanol, molecular and dissociative adsorption can occur on surface defects in TiO_2_ [46]. The existence of long-lived induced V_o_ as active sites after deposition of target molecules from methanolic solutions may be arguable, despite the expectedly short timeframes for the desorption of methanol from TiO_2_ in air. In addition, defects have been reported to accelerate the photocatalytic dissociation of methanol on TiO_2_, which has been attributed to a decrease of the dissociation reaction barrier [46,47].

## 4. Conclusion

Defected noble-metal-free TiO_2-*x*_ films have been compared as SERS and PIERS substrates, following different post-treatment methods to induce formation of oxygen vacancies (V_o_). The post-treatment approaches included UV-light irradiation (**UV-**TiO_2-*x*_), vacuum annealing (**VA-**TiO_2-*x*_) and argon etching (**AE-**TiO_2-*x*_). Raman band enhancement was demonstrated for every case, suggesting V_o_ formation played a key role in the enhancement mechanism. Relative EFs—with respect to SERS intensities—and V_o_ healing lifetimes upon exposure to air followed the same trend, i.e. **UV-**TiO_2-*x*_ < **AE-**TiO_2-*x*_ <** VA-**TiO_2-*x*_ despite the drastic reduction observed in **AE-**TiO_2-*x*_ films. This is in line with our previous observations correlating PIERS and photocatalytic efficiencies, where optimum (*goldilocks*) V_o_ densities (as in the case of **VA-**TiO_2-*x*_) were established in order to promote charge transport. The particular performance of **VA-**TiO_2-*x*_ films has been attributed to the formation of deep V_o_ sites in these films. Strategies towards the engineering of materials containing deep V_o_ sites (as those observed in [23]) may be highly effective for photocatalysis applications. Further considerations have also been discussed with attention to defect engineering strategies. It is important to highlight that the chemical enhancements described in this work are both substrate- and molecule-dependent and thus appropriate protocols will need to be established before PIERS can be used as a universal tool to evaluate photocatalytic materials.

## Data Availability

The data are provided in the electronic supplementary material [48].

## References

[1] Bell SEJ *et al.* 2020 Towards reliable and quantitative surface-enhanced Raman scattering (SERS): from key parameters to good analytical practice. Angew. Chem.– Int. Edn **59**, 5454-5462. (10.1002/anie.201908154) PMC7154527 31588641

[2] Tolaieb B, Constantino CJL, Aroca RF. 2004 Surface-enhanced resonance Raman scattering as an analytical tool for single molecule detection. Analyst **129**, 337-341. (10.1039/b312812a)

[3] Robinson AM, Harroun SG, Bergman J, Brosseau CL. 2011 Portable electrochemical surface-enhanced Raman spectroscopy system for routine spectroelectrochemical analysis. Anal. Chem. **84**, 1760-1764. (10.1021/ac2030078) 22242894

[4] Bell SEJ, Sirimuthu NMS. 2006 Surface-enhanced Raman spectroscopy (SERS) for sub-micromolar detection of DNA/RNA mononucleotides. J. Am. Chem. Soc. **128**, 15 580-15 581. (10.1021/ja066263w) 17147354

[5] Darby BL, Etchegoin PG, Le Ru EC. 2014 Single-molecule surface-enhanced Raman spectroscopy with nanowatt excitation. Phys. Chem. Chem. Phys. **16**, 23 895-23 899. (10.1039/C4CP03422H) 25277821

[6] Le Ru EC, Etchegoin PG. 2009 Principles of surface-enhanced Raman spectroscopy. Principles of Surface-Enhanced Raman Spectroscopy [Internet]. [cited 2023 Feb 9]. See https://shop.elsevier.com/books/principles-of-surface-enhanced-raman-spectroscopy/le-ru/978-0-444-52779-0.

[7] Sharma B, Frontiera RR, Henry AI, Ringe E, Van Duyne RP. 2012 SERS: materials, applications, and the future. Mater. Today. **15**, 16-25. (10.1016/S1369-7021(12)70017-2)

[8] Botti S, Cantarini L, Almaviva S, Puiu A, Rufoloni A. 2014 Assessment of SERS activity and enhancement factors for highly sensitive gold coated substrates probed with explosive molecules. Chem. Phys. Lett. **592**, 277-281. (10.1016/j.cplett.2013.12.063)

[9] Schatz GC, Young MA, Van Duyne RP. 2006 Electromagnetic mechanism of SERS. Physics and Applications. **103**, 19-46.

[10] Morton SM, Ewusi-Annan E, Jensen L. 2009 Controlling the non-resonant chemical mechanism of SERS using a molecular photoswitch. Phys. Chem. Chem. Phys. **11**, 7424-7429. (10.1039/b904745j) 19690714

[11] Langer J *et al.* 2020 Present and future of surface-enhanced Raman scattering. ACS Nano **14**, 28-117. (10.1007/3-540-33567-6_2) 31478375 PMC6990571

[12] Tognalli NÃ¡G, Cortés E, Hernández-Nieves AD, Carro P, Usaj G, Balseiro CA, Vela ME, Salvarezza RC, Fainstein A. 2011 From single to multiple Ag-layer modification of Au nanocavity substrates: a tunable probe of the chemical surface-enhanced Raman scattering mechanism. ACS Nano **5**, 5433-5443. (10.1021/nn200567m) 21675769

[13] Ben-Jaber S, Peveler WJ, Quesada-Cabrera R, Cortés E, Sotelo-Vazquez C, Abdul-Karim N, Maier SA, Parkin IP. 2016 Photo-induced enhanced Raman spectroscopy for universal ultra-trace detection of explosives, pollutants and biomolecules. Nat. Commun. **7**, 12189. (10.1038/ncomms12189) 27412699 PMC4947161

[14] Mezhenny S, Maksymovych P, Thompson TL, Diwald O, Stahl D, Walck SD, Yates JT. 2003 STM studies of defect production on the TiO_2_ (110)-(1 × 1) and TiO_2_ (110)-(1 × 2) surfaces induced by UV irradiation. Chem. Phys. Lett. **369**, 152-158. (10.1016/S0009-2614(02)01997-8)

[15] Henrich VE, Dresselhaus G, Zeiger HJ. 1976 Observation of two-dimensional phases associated with defect states on the surface of TiO_2_. Phys. Rev. Lett. **36**, 1335. (10.1103/PhysRevLett.36.1335)

[16] Dagdeviren OE, Glass D, Sapienza R, Cortés E, Maier SA, Parkin IP, Grütter P, Quesada-Cabrera R. 2021 The effect of photoinduced surface oxygen vacancies on the charge carrier dynamics in TiO_2_ films. Nano Lett. **21**, 8348-8354. (10.1021/acs.nanolett.1c02853) 34582208

[17] Glass D *et al.* 2019 Dynamics of photo-induced surface oxygen vacancies in metal-oxide semiconductors studied under ambient conditions. Adv. Sci. **6**, 1901841. (10.1002/advs.201901841) PMC6864511 31763155

[18] Zhao J *et al.* 2021 Recent advances and perspectives in photo-induced enhanced Raman spectroscopy. Nanoscale **13**, 8707-8721. (10.1039/D1NR01255J) 33960340

[19] Almohammed S, Zhang F, Rodriguez BJ, Rice JH. 2018 Photo-induced surface-enhanced Raman spectroscopy from a diphenylalanine peptide nanotube-metal nanoparticle template. Sci. Rep. **8**, 3880. (10.1038/s41598-018-22269-x) 29497167 PMC5832858

[20] Al-Shammari RM, Baghban MA, Al-Attar N, Gowen A, Gallo K, Rice JH, Rodriguez BJ. 2018 Photoinduced enhanced Raman from lithium niobate on insulator template. ACS Appl. Mater. Interfaces **10**, 30 871-30 878. (10.1021/acsami.8b10076) 30107124

[21] Fularz A, Almohammed S, Rice JH. 2020 Oxygen incorporation-induced SERS enhancement in silver nanoparticle-decorated ZnO nanowires. ACS Appl. Nano Mater. **3**, 1666-1673. (10.1021/acsanm.9b02395)

[22] Abid K, Belkhir NH, Jaber SB, Zribi R, Donato MG, Di Marco G, Gucciardi PG, Neri G, Maâlej R. 2020 Photoinduced enhanced raman spectroscopy with hybrid Au@WS2 nanosheets. J. Phys. Chem. C **124**, 20 350-20 358. (10.1021/acs.jpcc.0c04664)

[23] Brognara A, Bricchi BR, William L, Brinza O, Konstantakopoulou M, Bassi AL, Ghidelli M, Lidgi-Guigui N. 2022 New mechanism for long photo-induced enhanced Raman spectroscopy in Au nanoparticles embedded in TiO_2_. Small **18**, 2201088. (10.1002/smll.202201088) 35616163

[24] Glass D *et al.* 2021 Probing the role of atomic defects in photocatalytic systems through photoinduced enhanced Raman scattering. ACS Energy Lett. **6**, 4273-4281. (10.1021/acsenergylett.1c01772)

[25] Ren Q, He Y, Wang H, Sun Y, Dong F. 2022 Photo-switchable oxygen vacancy as the dynamic active site in the photocatalytic NO oxidation reaction. ACS Catal. **12**, 14 015-14 025. (10.1021/acscatal.2c03353)

[26] Lee Y, Kim H, Lee J, Yu SH, Hwang E, Lee C, Ahn J-H, Cho JH. 2015 Enhanced Raman scattering of rhodamine 6G films on two-dimensional transition metal dichalcogenides correlated to photoinduced charge transfer. Chem. Mater. **28**, 180-187. (10.1021/acs.chemmater.5b03714)

[27] Jiang L, You T, Yin P, Shang Y, Zhang D, Guo L, Yang S. 2013 Surface-enhanced Raman scattering spectra of adsorbates on Cu_2_O nanospheres: charge-transfer and electromagnetic enhancement. Nanoscale **5**, 2784-2789. (10.1039/c3nr33502j) 23435689

[28] Cong S *et al.* 2015 Noble metal-comparable SERS enhancement from semiconducting metal oxides by making oxygen vacancies. Nat. Commun. **6**, 7800. (10.1038/ncomms8800) 26183467 PMC4518302

[29] Musumeci A, Gosztola D, Schiller T, Dimitrijevic NM, Mujica V, Martin D, Rajh T. 2009 SERS of semiconducting nanoparticles (TiO_2_ hybrid composites). J. Am. Chem. Soc. **131**, 6040-6041. (10.1021/ja808277u) 19364105

[30] Yang L, Jiang X, Ruan W, Zhao B, Xu W, Lombardi JR. 2008 Observation of enhanced Raman scattering for molecules adsorbed on TiO_2_ nanoparticles: charge-transfer contribution. J. Phys. Chem. C **112**, 20 095-20 098. (10.1021/jp8074145)

[31] Hugenschmidt MB, Gamble L, Campbell CT. 1994 The interaction of H_2_O with a TiO_2_(110) surface. Surf. Sci. **302**, 329-340. (10.1016/0039-6028(94)90837-0)

[32] Guillemot F, Porté MC, Labrugère C, Baquey C. 2002 Ti^4+^ to Ti^3+^ conversion of TiO_2_ uppermost layer by low-temperature vacuum annealing: interest for titanium biomedical applications. J. Colloid Interface Sci. **255**, 75-78. (10.1006/jcis.2002.8623) 12702370

[33] Song G, Cong S, Zhao Z. 2022 Defect engineering in semiconductor-based SERS. Chem. Sci. **13**, 1210-1224. (10.1039/D1SC05940H) 35222907 PMC8809400

[34] Wang R, Hashimoto K, Fujishima A, Chikuni M, Kojima E, Kitamura A, Shimohigoshi M, Watanabe T. 1998 Photogeneration of highly amphiphilic TiO_2_ surfaces. Adv. Mater. **10**, 135-138. (10.1002/(SICI)1521-4095(199801)10:2<135::AID-ADMA135>3.0.CO;2-M)

[35] Bharti B, Kumar S, Lee HN, Kumar R. 2016 Formation of oxygen vacancies and Ti^3+^ state in TiO_2_ thin film and enhanced optical properties by air plasma treatment. Sci. Rep. **6**, 32355. (10.1038/srep32355) 27572095 PMC5004114

[36] Hüttenhofer L, Eckmann F, Lauri A, Cambiasso J, Pensa E, Li Y, Cortes E, Sharp ID, Maier SA. 2020 Anapole excitations in oxygen-vacancy-rich TiO_2–x_ nanoresonators: tuning the absorption for photocatalysis in the visible spectrum. ACS Nano **14**, 2456-2464. (10.1021/acsnano.9b09987) 31995353

[37] Kurtz RL, Stock-Bauer R, Msdey TE, Román E, De Segovia JL. 1989 Synchrotron radiation studies of H_2_O adsorption on TiO_2_(110). Surf. Sci. **218**, 178-200. (10.1016/0039-6028(89)90626-2)

[38] Onda K, Li B, Petek H. 2004 Two-photon photoemission spectroscopy of TiO_2_ (110) surfaces modified by defects and O_2_ or H_2_O adsorbates. Phys. Rev. B **70**, 45415. (10.1103/PhysRevB.70.045415)

[39] Pristinski D, Tan S, Erol M, Du H, Sukhishvili S. 2006 *In situ* SERS study of Rhodamine 6G adsorbed on individually immobilized Ag nanoparticles. J. Raman Spectrosc. **37**, 762-770. (10.1002/jrs.1496)

[40] Wang J *et al.* 2022 Subsurface engineering induced fermi level de-pinning in metal oxide semiconductors for photoelectrochemical water splitting. Angew. Chem. Int. Ed. **135**, e202217026. (10.1002/anie.202217026) 36577697

[41] Setvin M, Shi X, Hulva J, Simschitz T, Parkinson GS, Schmid M, Di Valentin C, Selloni A, Diebold U. 2017 Methanol on anatase TiO_2_ (101): mechanistic insights into photocatalysis. ACS Catal. **7**, 7081-7091. (10.1021/acscatal.7b02003) 29034122 PMC5634753

[42] Berger T, Sterrer M, Diwald O, Knözinger E, Panayotov D, Thompson TL, Yates JT. 2005 Light-induced charge separation in anatase TiO_2_ particles. J. Phys. Chem. B **109**, 6061-6068. (10.1021/jp0404293) 16851666

[43] Shondo J, Veziroglu S, Tjardts T, Sarwar TB, Mishra YK, Faupel F, Aktas OC. 2022 Nanoscale synergetic effects on Ag–TiO_2_ hybrid substrate for photoinduced enhanced Raman spectroscopy (PIERS) with ultra-sensitivity and reusability. Small **18**, 2203861. (10.1002/smll.202203861) 36135727

[44] Bikondoa O, Pang CL, Ithnin R, Muryn CA, Onishi H, Thornton G. 2006 Direct visualization of defect-mediated dissociation of water on TiO_2_(110). Nat. Mater. **5**, 189-192. (10.1038/nmat1592)

[45] Feng J *et al.* 2019 Surface-bound sacrificial electron donors in promoting photocatalytic reduction on titania nanocrystals. Nanoscale **11**, 19 512-19 519. (10.1039/C9NR05453G) 31573006

[46] Zhang Z, Bondarchuk O, White JM, Kay BD, Dohnálek Z. 2006 Imaging adsorbate O−H bond cleavage: methanol on TiO_2_(110). J. Am. Chem. Soc. **128**, 4198-4199. (10.1021/ja058466a) 16568973

[47] Mao X, Wang Z, Lang X, Hao Q, Wen B, Dai D, Zhou C, Liu L-M, Yang X. 2015 Effect of surface structure on the photoreactivity of TiO_2_. J. Phys. Chem. C **119**, 6121-6127. (10.1021/acs.jpcc.5b00503)

[48] Ben-Jaber S, Glass D, Brick T, Maier SA, Parkin IP, Cortés E, Peveler WJ, Quesada-Cabrera R. 2023 Photo-induced enhanced Raman spectroscopy as a probe for photocatalytic surfaces. Figshare. (10.6084/m9.figshare.c.6777781) PMC10493551 37691466

